# A Protein Kinase Cε/Protein Kinase D3 Signalling Axis Modulates RhoA Activity During Cytokinesis

**DOI:** 10.3390/biomedicines13020345

**Published:** 2025-02-03

**Authors:** Ursula Braun, Michael Leitges

**Affiliations:** Division of BioMedical Sciences, Faculty of Medicine, Memorial University of Newfoundland, 300 Prince Philip Drive, St. John’s, NL A1B 3V6, Canada

**Keywords:** protein kinase D3, protein kinase Cε, RhoA, gene targeting, mouse embryonic fibroblasts, cytokinesis

## Abstract

Background: Protein kinase D3 (PKD3) is a member of the PKD family that has been implicated in many intracellular signalling pathways. However, defined statements regarding PKD isoform specificity and in vivo functions are rare. Methods: Here, we use PKD3-depleted mouse embryonic fibroblast cells and employ various cell culture-based assays and fluorescence microscopy. Results: We show that PKD3 is involved in the regulation of cytokinesis after immortalisation by modulating RhoA activity through a PKCε/PKD3 signalling axis. Conclusions: PKD3 depletion leads to prolonged RhoA activity during cytokinesis, resulting in failed abscission and an increase in the number of multinucleated cells. This identifies a novel, previously unrecognised PKCε/PKD3 pathway involved in the modulation of cytokinesis.

## 1. Introduction

The protein kinase D (PKD) family of Ser/Thr-specific kinases consists of three members, designated PKD1–3. Based on their structural elements, PKDs are linked to the diacylglycerol-mediated signalling pathway, which is involved in multiple functions and defines them as multifunctional kinases [1], and are currently classified as members of the CaM family of calcium/calmodulin-dependent kinases [2]. In particular, PKD3, originally isolated as PKCν [3], has been implicated in many intercellular signalling pathways. However, isoform-specific data on the in vivo functions of individual PKD family members remain very limited. PKD activation is thought to be mediated by transphosphorylation at serine residues in the activation loop by novel PKC isoforms [4]. PKCs co-localise to lipid membranes, providing a link between the PKC and PKD signalling cascades. Following the activation loop phosphorylation, the C-terminal Ser 916 (in mouse) becomes autophosphorylated [5]. Noteworthy is that all PKD isoforms contain an activation loop motif, whereas only PKD1 and 2 contain the C-terminal autophosphorylation motif. This indicates that certain activation mechanisms in PKD3 are different from the rest of the family. Both phosphorylation events have been established as markers of the activation status of PKDs. However, recent research has also identified a signalling pathway that activates PKDs in the absence of PKC activity, reviewed in [6]. Nevertheless, the PKC/PKD axis is an established signalling cascade of PKD-mediated signalling [7]. In particular, PKCε has been identified as an activating kinase of PKD [8]. Cytokinesis is required to ensure the establishment of two equal daughter cells and is characterised by the presence of a cleavage furrow, reviewed in [9], which is established by actin filaments and non-muscle myosin II assembling a contractile ring at the equatorial position of the cell cortex. During ingression, the furrow moves into the midzone. Once a midbody is formed, the furrow process stops and abscission is required to complete cytokinesis and promote the generation of two daughter cells. The small GTPase RhoA, activated by specific guanine nucleotide exchange factors (GEFs), has been implicated in this process, including the establishment of the cleavage furrow [10]. The coordinated inactivation of RhoA by a specific GTPase-activating protein (GAP) has also been shown to be important for the timely depolymerisation of the furrow structure at the end of cytokinesis and for accurate abscission. Recent work suggests that PKCε plays a key role in regulating RhoA-mediated events in cytokinesis by controlling the timely exit of RhoA from the midbody zone. It has been shown that PKCε translocates to the midbody and drives the local downregulation of RhoA activity. This event eventually leads to the depletion of active RhoA from this zone and subsequently causes actin depolarisation, a prerequisite for abscission [11]. In the present study, we identified PKD3 as a PKCε substrate and confirmed its role in regulating RhoA activity during the late phase of cytokinesis.

## 2. Materials and Methods

### 2.1. Generation of a Mutant PRKD3 Allele in Mice

To clone a targeting vector for the PRKD3 gene locus, we obtained a BAC clone from Gene Service (www.geneservice.co.uk, accssed on 1 January 2008) and identified the desired sequence using the Ensembl gene browser. This BAC contained the 2nd and 3rd exons of the PRKD3 gene (clone ID: bMQ-135M20). After verifying the sequence of the obtained BAC clone, we used recombineering tools and standard strategies to clone a targeting vector for the PRKD3 gene locus. The final construct contained a single LoxP site 5′ of the 2nd exon and a second LoxP site 3′ of the 3rd exon. This first site was immediately followed by a neomycin expression cassette flanked by Frt sites. Sequential incubation with the Flp and the Cre recombinase is predicted to result in a deletion of an approximately 5.2 kb genomic fragment, including exons 2 and 3, causing a frame shift within the transcript and leading to nonsense mRNA. In total, the generated targeting vector contained a 9.9 kb genomic sequence of the PRKD3 locus. Prior to electroporation, the targeting vector was linearised with NotI at the 5′ end of the genomic sequence. Embryonic stem (ES) cells from the E14.1 substrain (129/Ola background) were electroporated with 40 μg of linearised vector and selected for G418 (350 μg/mL) resistance for 10 days. Electroporation conditions were applied as followed: 240 V, cuvettes with 4 mm electrode distance and 480 μF capacitance. From two independent electroporations, at least 2 × 96 resistant clones per electroporation were screened for homologous recombination of the targeting vector by Southern blot analysis. An endogenous probe (5′ probe—see Supplementary Figure S1) was used to identify an 18.6 kb fragment in the wild type in addition to a 4.5 kb fragment after homologous recombination. Positive clones were then further characterised for correct and single integration of the targeting vector using different probes and restriction enzymes (as indicated in Supplementary Figure S1). Beside the 5′ and 3′ probe, we also applied an internal neo probe for further verification of the clones. The target frequency observed was approximately 1%. Two verified independent ES cell clones were then used for injections into C57Bl/6 blastocysts. Chimeric males were produced for both clones. Males with more than 50% chimerism, analysed by the patched coat colors, were then mated to C57Bl/6 females to test for germline transmission. Both lines produced F1 heterozygous males, which were immediately crossed to a ubiquitously expressing Flp transgenic mouse line to delete the neomycin cassette. The success of this deletion was confirmed by neomycin-specific polymerase chain reaction (PCR) and Southern blot analysis using the neomycin gene as a probe. The resulting mouse line represents a floxed PRKD3 allele (PKD3fl/+) that can be used for tissue- and cell-specific deletion analysis using Cre transgenic animals. For this study, we crossed the PKD3fl/+ mice with a ubiquitous Cre-expressing transgenic line to obtain a deleted allele (PKD3Δ/+). Again, the functionality of the LoxP sites was confirmed by Southern blot analysis and a specific PCR assay. Heterozygous PKD3Δ/+ breeding pairs were then used to establish a homozygous PKD3Δ/Δ mouse line for initial analysis. For standard genotyping, genomic DNA was prepared from ear tag biopsies and used as a template in a specific PCR using the following primers listed in Table 1.

Primer pair a/b gave a 458 bp wild-type fragment and a 593 bp recombinant fragment in the presence of an integrated LoxP site and was used to identify the PKD3fl/+ allele. Primer pair c/d gave a 488 bp wild-type fragment, which only occurs in the absence of deletion, whereas primer pair e/f gave a deletion-specific 277 bp fragment, which was used to identify the PKD3Δ/+ allele.

### 2.2. Isolation of MEFs, Immortalisation and Cell Culture

Heterozygous matings of PKD3Δ/+ animals were used to isolate mouse embryonic fibroblasts (MEFs) expressing the PKD3 mutant allele. Embryos at 12.5 to 13.5 postcoitum were used for preparation according to standard protocols [12]. A modified NIH 3T3 protocol was used to immortalise MEFs. MEFs were cultured in Gibco DMEM+ GlutaMAXTM-I (Invitrogen, Waltham, MA, USA) medium supplemented with 10% Gibco fetal calf serum (Invitrogen), Gibco non-essential amino acids (Invitrogen), penicillin (50 units/mL) and streptomycin (100 μg/mL) (Invitrogen).

### 2.3. RhoA Pull Down

The active RhoA pull down and detection kit was purchased from Thermo Scientific (Waltham, MA, USA) (Cat. 16116). Protein extraction and active RhoA pull down were performed according to the manufacturer’s protocol.

### 2.4. Cell Fixation, Permeabilisation and Immunostaining

MEFs were grown on coverslips to 70–80% confluence and fixed with 4% paraformaldehyde in PBS for 15 min at room temperature. Fixed cells were then permeabilized with 0.1% saponin (Sigma-Aldrich, St. Louis, MO, USA, Cat. 8047-15-2) in PBS for 15 min and blocked with PBS containing 20 mM glycine, 0.1% saponin and 3% BSA for 15 min at room temperature. Primary antibodies were applied in PBS containing 0.1% saponin and 3% BSA and incubated overnight at 4 °C with slow rotation. Secondary antibodies were applied in PBS containing 0.1% saponin and 3% BSA for varying periods from 2 h (at RT) to overnight (at 4 °C). Fluorophore-conjugated primary antibodies were applied in PBS containing 0.1% saponin and 3% BSA for varying times from 2 h (at RT) to overnight (at 4 °C). Alternatively, for RhoA detection in MEFs without GFP or cherry fusion protein expression, MEFs were fixed in 10% tri-chloroacitic acid (TCA) for 10 min at room temperature. The fixed cells were then permeabilized with 0.3% Triton PBS for 10 min at room temperature and blocked with PBS containing 30 mM glycine, 1% BSA and 10% sheep serum for 1 h. Primary antibodies were then applied in PBS containing 30 mM glycine, 3% sheep serum and 1% BSA and incubated overnight at 4 °C.

### 2.5. Confocal Imaging and Processing

DAPI-containing mounting medium (ProLong antifade reagent with DAPI, Invitrogen, Cat. 36935) was used to mount the coverslips for confocal microscopy. MEFs were visualised using a Zeiss LSM 510 Meta inverted microscope equipped with a Zeiss LSM laser module purchased from ZEISS AG (Oberkochen, Germany). Images were captured using an AxioCam HMr digital camera purchased from ZEISS AG (Oberkochen, Germany). The images were processed and enhanced using Carl Zeiss free software ZEN (2008).

### 2.6. Quantification of Double-Nucleated Cells

MEF cells were stained with phalloidin and mounted with mounting medium containing DAPI and then visualised at 20× magnification using a Zeiss LSM510 Meta inverted microscope, followed by image capture. For wt, PKCε^-/-^ and PKD3^-/-^ MEFs, the sample size was 1000 cells (n = 3). For various PKD3-GFP and constructs expressing MEFs, the sample size was 200 cells (n = 3). The data were analysed using Microsoft Excel (2010).

### 2.7. Quantification of RhoA^+^/RhoA^−^ Cells

MEFs were stained with a RhoA antibody conjugated to Alexa Fluor^®^ 488 (Santa Cruz, Dallas, TX, USA, sc-418) and phalloidin and mounted with DAPI containing mounting medium. Images were captured using a Zeiss LSM510 Meta inverted microscope at 63× magnification. Cytokinesis-dividing cells (identified by the presence of a cleavage furrow) from a 70% confluent 14 mm diameter coverslip were counted (n = 6). Each sample contained an average of 30–40 cells undergoing cytokinesis. Among these, RhoA^+^ (expressing a RhoA signal overlapping the cleavage furrow) and RhoA^−^ (expressing no RhoA signal overlapping the cleavage furrow) cells were counted separately. Statistical analysis was performed using Microsoft Excel (2010).

### 2.8. Generation of PKD Overexpression Constructs

MEF lines: To overexpress individual PKD1-3 isoforms in wild-type, PKD3^-/-^ and PKCε^-/-^ MEFs, the pLenti6/TR vector system was used according to the manufacturer’s instructions obtained from Invitrogen (catalogue number V480-20). To use the Gateway system (Invitrogen) for subcloning, mouse cDNAs for each PKD isoform were subcloned into the pENTRTM/D-TOPO vector (Invitrogen, Cat. No. K2400-20). The QuickChange II XL site-directed mutagenesis kit (Agilent Technologies, Santa Clara, CA, USA, Cat. No. 200521) was used to introduce the indicated mutations according to the manufacturer’s instructions. The individual mutagenesis primers are listed in Table 2.

### 2.9. Antibodies Used

PKD/PKCμ (#2052), phosphoPKD Ser744/Ser748 (#2054S), PKD3/PKCν (#2054L) and GAPDH (#2118) were purchased from Cell Signaling (Danvers, MA, USA). mCherry (ab167453) was purchased from Abcam (Cambridge, UK). PKD2 (E-20, sc-74839), RhoA (26C4) Alexa Fluor^®^ 488 and Alexa Fluor^®^ 647 (sc-418) were purchased from Santa Cruz (Dallas, TX, USA). Goat anti-rabbit HRPO antibody (#110449) was purchased from Jackson Immunoresearch Labs (West Grove, PA, USA). Alexa Fluor^®^ 647 goat anti-rabbit IgG (H+L) antibody (A22287), Alexa Fluor^®^ donkey anti-rabbit IgG (H+L) antibody (A10042) and Alexa Fluor^®^ 647 phalloidin (A22287) were purchased from Life Technology (Carlsbad, CA, USA).

## 3. Results

### 3.1. Generation of a PKD3 Null Allele in Mice and Isolation and Characterisation of Mouse Embryonic Fibroblasts (MEFs)

**Figure 1:**
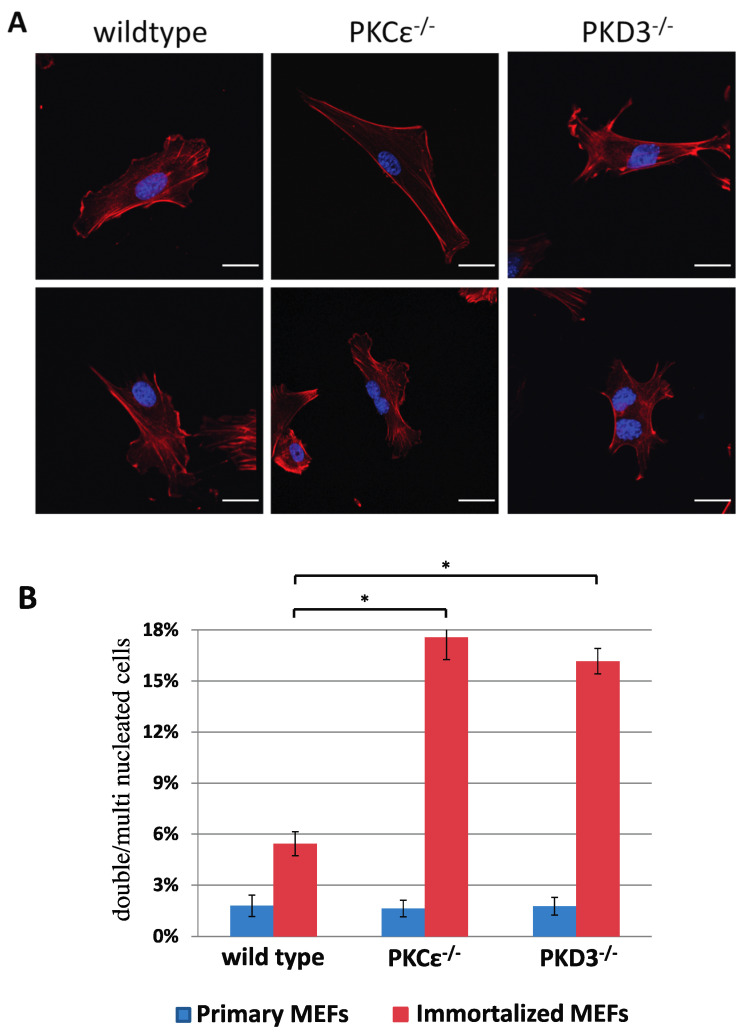
Double/multi-nucleation in immortalized PKCε- and PKD3-deficient MEFs. (**A**) MEFs of the indicated genotypes were fixed with 4% PFA and stained with phalloidin and DAPI. The top row shows representative images of primary MEFs (passage number 2–4), and the second row shows representative images of MEFs after immortalisation (>10 passages). The experimental details are described in Section 2. Scale bar, 24 μm. (**B**) The graph shows a calculation of single-nucleated versus double/multi-nucleated MEFs of various genotypes and compares primary with immortalized cells. The genotypes are indicated below, the primary values are shown in blue, the immortalized values are shown in red and the error bars represent the standard deviation; * indicates *p*-values 0.000072 for wt/PKCε^-/-^ and 0.000028 for wt/PKD3^-/-^ by *t*-test.

To identify and analyse the in vivo functions of PKD3, we used a standard gene targeting approach (see the Materials and Methods section and Supplementary Figure S1 for details) to generate a conditional homozygous PKD3 mouse line (PKD3fl/fl) and a subsequent homozygous PKD3^-/-^ mouse line. Heterozygous intercrosses of PKD3^+/-^ mice revealed a change in the expected Mendelian ratio from 25 to 12% of the PKD3^-/-^ genotype (a total of 117 progeny were genotyped, resulting in 36 wild-type [31%], 67 heterozygous [67%] and 14 homozygous [12%] individuals). The specific reason for this observed partial embryonic lethality was not clear but will be analysed in the future. Apart from this phenomenon, PKD3-deficient animals appeared normal and did not express any obvious dramatic phenotypes within the first 6–9 months of life, except that both sexes were less productive than the corresponding wild-type animals. To characterise whether altered signalling events occur as a result of PKD3 depletion, we established primary and immortalised MEFs from PKD3-deficient and corresponding wild-type embryos. While primary MEFs from both genotypes showed no differences in growth performance or morphology, immortalised PKD3^-/-^ MEFs were shown to harbour a high content of double and multi-nucleated cells under normal cell culture conditions (Figure 1A,B). As a similar phenomenon has been described following PKCε siRNA knockdown in HeLa cells [11], wild-type and PKCε^-/-^ MEFs were also included in this study. Indeed, primary, immortalised PKCε^-/-^ and PKD3^-/-^ MEFs were characterised with up to 17% of double/multi-nucleated cells (Figure 1B) compared to wild-type MEFs (up to 5%), suggesting that PKCε and PKD3 may act in a similar pathway.

### 3.2. PKD3 Localises to the Furrow During Cytokinesis

**Figure 2:**
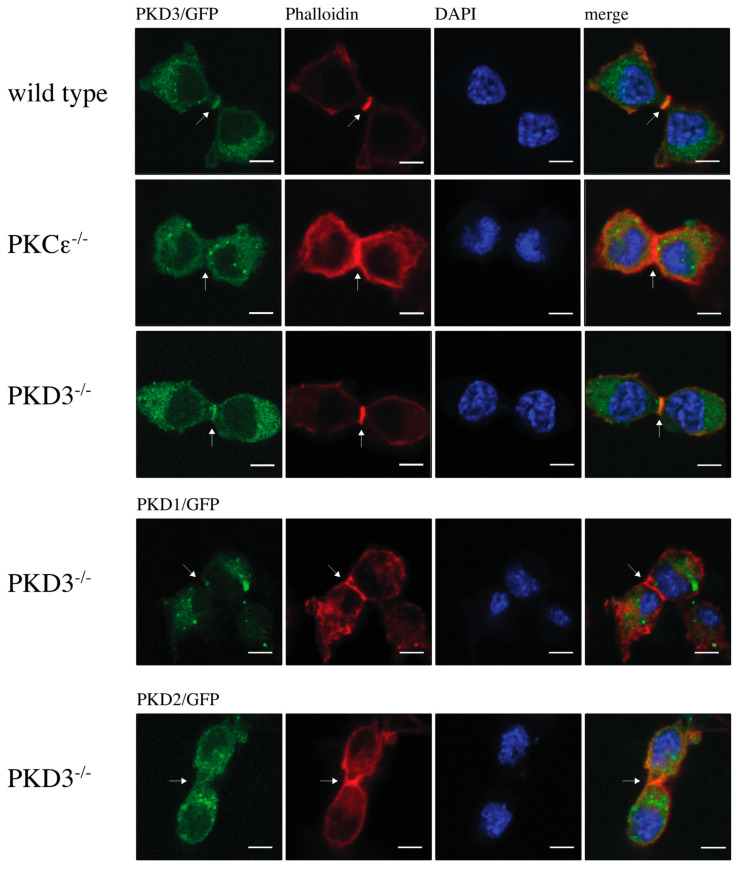
Localisation of protein kinase D during cytokinesis: The top row shows wild-type MEFs expressing PKD3/GFP, the 2nd row shows PKCε^-/-^ MEFs expressing PKD3/GFP, the 3rd row shows PKD3^-/-^ MEFs expressing PKD3/GFP, and the 4th and 5th rows show PKD3^-/-^ MEFs expressing either PKD1/GFP or PKD2/GFP as indicated. All MEFs were fixed with 4% PFA and stained with phalloidin and DAPI. All experimental details are described in Section 2. Representative images of MEFs during cytokinesis are indicated by the presence of the cleavage furrow. The arrows indicate the position of the cleavage furrow, and the expression of the exogenous proteins is shown in Supplementary Figure S2; scale bar, 48 μm.

The double/multi-nucleated phenotype observed in PKCε-depleted cells has been linked to its role in cytokinesis [11]. Therefore, we hypothesised that the localisation of PKD3 during cytokinesis might support a possible functional association with PKCε. Indeed, we observed that GFP-labelled PKD3 localised to the furrow and overlapped with the F-actin ring (Figure 2, top row). Interestingly, the localisation of PKD3 to the furrow was shown to be PKCε-dependent (Figure 2, second top row). The reintroduction of PKD3 into PKD3^-/-^ MEFs by lentivirus-based expression of PKD3/GFP restored PKD3 localisation to the furrow (Figure 2, middle row) and rescued the double/multi-nucleated phenotype To determine whether furrow localisation is an isoform-specific function, PKD1 and PKD2-GFP constructs were introduced into PKD3^-/-^ MEFs and localisation and nucleation were assessed. Neither PKD1 nor PKD2 expression was sufficient to rescue cleavage furrow-specific localisation during cytokinesis (Figure 2, bottom two rows) or the double/multi-nucleated phenotype. Taken together, these data suggest that PKCε is required for cleavage furrow-specific PKD3 localisation and highlight a non-redundant function of PKD3.

### 3.3. Dominant-Active and -Inactive Mutations of PKD3 Affect Wild-Type and PKCε^-/-^ MEFs During Cytokinesis

**Figure 3:**
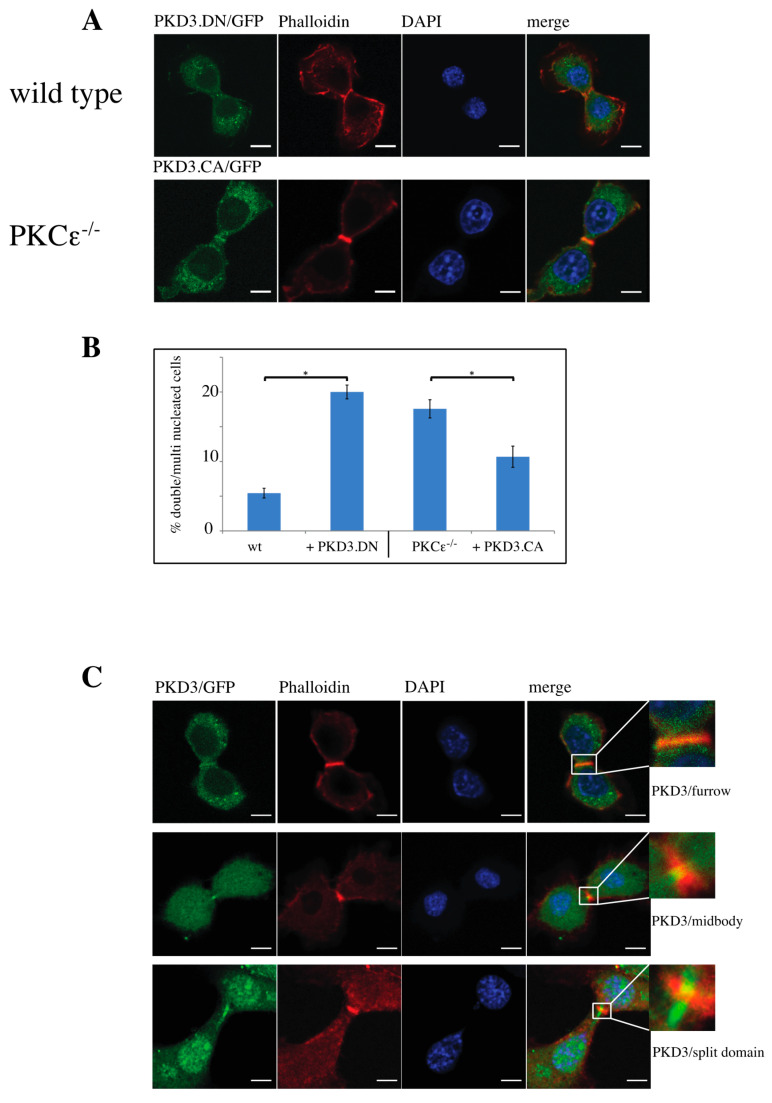
Characterisation of functional PKD3 mutations and their dynamic behavior during cytokinesis: (**A**) The top row depicts wild-type MEFs expressing a PKD3.DN/GFP construct, while the second row shows PKCε^-/-^ MEFs expressing a PKD3.CA/GFP construct. MEFs of the indicated genotypes were fixed with 4% PFA and stained with phalloidin and DAPI. All experimental details are described in Section 2. Representative images of MEFs during cytokinesis are indicated by the presence of the cleavage furrow. (**B**) The graph shows a calculation of single-nucleated versus double/multi-nucleated MEFs of the wild-type and PKCε^-/-^ genotype expressing either PKD3.DN or PKD3.CA constructs, as indicated. The error bars represent the standard deviation; * indicates *p*-values 0.002257 for wt/PKD3.DN and 0.004046 for PKCε^-/-^/PKD3.CA by *t*-test. (**C**) Representative images of wild-type MEFs expressing PKD3/GFP at various stages during cytokinesis are shown. The top row indicates an early phase of cytokinesis with a cleavage furrow staining of PKD3, the middle row demonstrates a midbody staining of PKD3 and the bottom row shows a late stage of cytokinesis with a split PKD3 domain at the midbody. The higher-magnification inset shows the cleavage furrow area in detail; scale bar, 48 μm.

**Figure 4:**
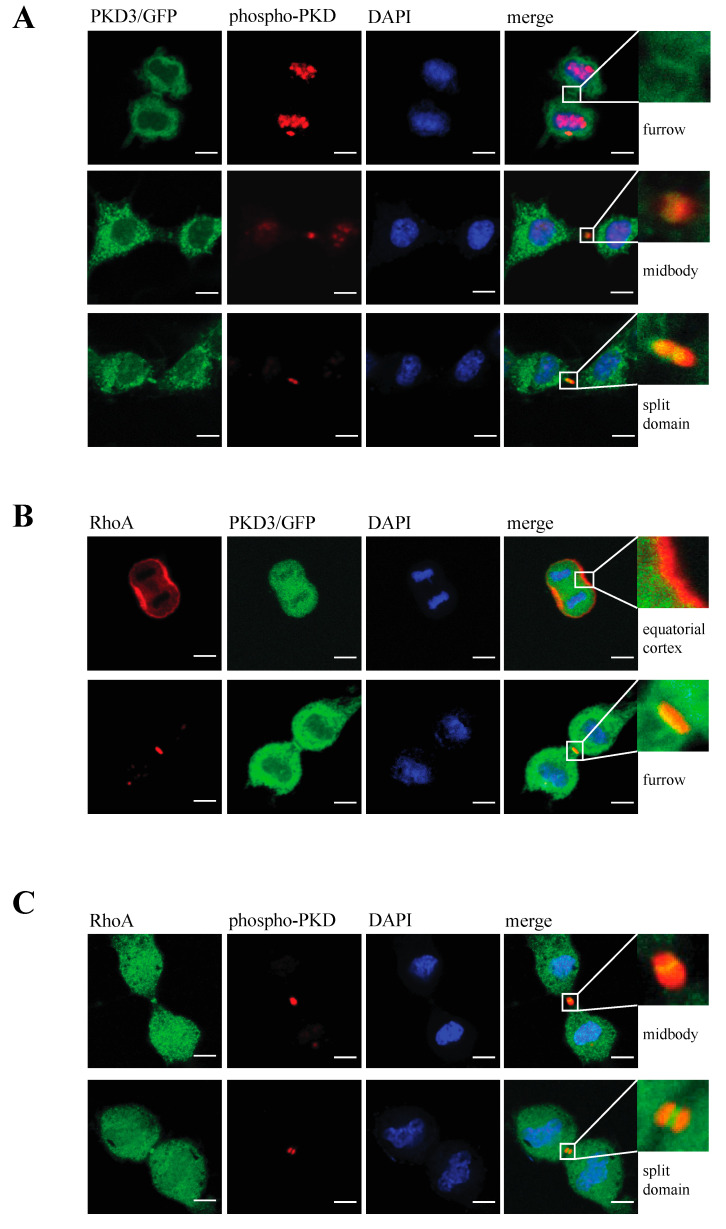
Analysis of PKD3 activity in correlation to PKD3 and RhoA localisation during cytokinesis: (**A**) Cells expressing PKD3/GFP were fixed with 4% PFA and co-stained with an anti-phospho Ser 730/734 PKD antibody and DAPI at various stages of the cytokinesis. The top row represents an early stage of cytokinesis with PKD3 localized at the newly established cleavage furrow. The middle row indicates PKD3/GFP as a single spot at the midbody area overlapping with the phospho-PKD signal. The bottom row depicts late -stage cytokinesis with a split PKD3/GFP signal at the midbody area that overlaps with the phospho-PKD signal. All higher magnification inserts represent the furrow area of the corresponding merge, as indicated. (**B**) Cells expressing PKD3/GFP were fixed with 1% TCA and co-stained with an anti-RhoA antibody and DAPI. The first row represents a very early stage of cytokinesis, while the second row shows a more progressed stage when a cleavage furrow has already been established. Higher magnification of the first row shows the equatorial cortex, while the cleavage furrow is shown in the second blow up. (**C**) Wild-type MEFs were co-stained with anti-RhoA and anti-phospho-PKD antibodies. The top row indicates a stage when phospho-PKD is localized at the midbody, and the bottom row represents a stage when the phospho-PKD signal is split at the midbody. The high-magnification inserts represent the furrow area of the corresponding merge, as indicated. Scale bar, 48 μm.

Next, we determined whether the activation status of PKD3 has an effect on its specific localisation during cytokinesis and the subsequent multinucleation phenotype. To address this question, we generated an inactive PKD3 construct by mutating both serine residues in the activation loop to alanines (in mouse: Ser 730/734; PKD3.DN/GFP) and a constitutively activated version of PKD3 by mutating the same residues to glutamate (PKD3.CA/GFP). Indeed, expression of the PKD3.DN/GFP construct in wild-type MEFs resulted in a loss of specific furrow localisation (Figure 3A, top row) and a significant increase in the percentage of double/multi-nucleated cells (Figure 3B). Consistent with these results, expression of the PKD3.CA/GFP mutant in PKCε^-/-^ MEFs partially rescued the original phenotype associated with specific furrow localisation during cytokinesis (Figure 3A,B). We therefore concluded that PKCε, via its potential ability to phosphorylate PKD3 at Ser 730/734 in the activation loop, may be involved either directly or indirectly in the localisation and subsequent activation of PKD3 during cytokinesis. During this analysis, we found that the localisation of PKD3/GFP to the furrow during cytokinesis appeared to be a dynamic process that started with overlapping furrow staining (Figure 3C, top row), was followed by midbody staining while the cleavage furrow was still established (Figure 3C, middle row) and ended with a split domain in the centre of the furrow oriented at 90 degrees to the furrow axis (Figure 3C, bottom row). To correlate the activation status of PKD3 with the three different staining patterns, we used an antibody that specifically recognises the phosphorylated serines within the activation loop of PKDs (including PKD3 at Ser 730/734 in mouse), which is widely used as a marker for activated PKD, to show that PKD3 was not phosphorylated when localised to the furrow at this early stage (Figure 4A, top row). Interestingly, PKD3 was shown to be phosphorylated at Ser 730/734 when first localised to the midbody as a single spot (Figure 4A, middle row), and during subsequent cleavage, the PKD3 domain showed overlap with the activity-specific phosphorylation signal (Figure 4A, bottom row). To verify the specificity of the active PKD signal, we also tested the phospho-specific antibody on PKD3-deficient MEFs. Indeed, PKD3^-/-^ MEFs lacked the specific signal at the furrow (Supplementary Figure S2A), while prominent staining remained in the newly formed nucleus. We attributed this signal to PKD1, as this isoform has previously been reported to localise to this compartment [13]. In addition, PKCε^-/-^ MEFs also lacked the furrow-specific activated PKD signal, suggesting that PKCε functions upstream of PKD3 in this context (Supplementary Figure S2A). Taken together, these data suggest that activated PKD3 is localised to the furrow during later stages of cytokinesis but prior to abscission and that activated PKD3 is restricted to an area at the midbody via a mechanism involving upstream PKCε signalling.

### 3.4. PKD3 Regulates the Activity but Not the Localisation of RhoA

**Figure 5:**
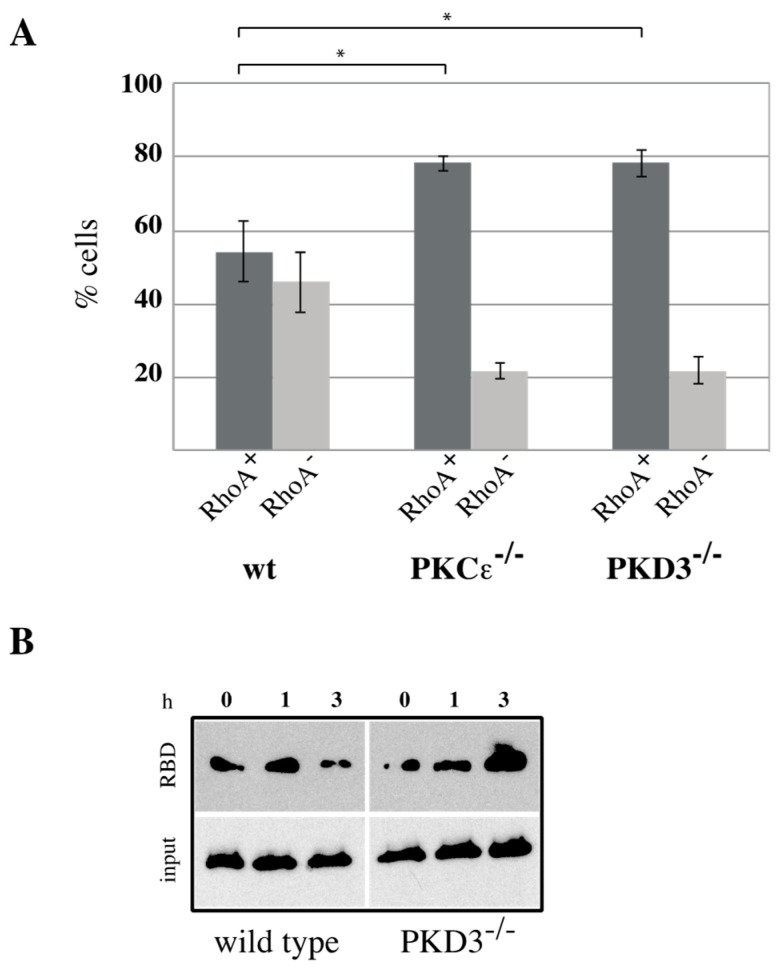
RhoA activity is altered in PKCε- and PKD3-deficient MEFs: (**A**) RhoA^+^ cells were analyzed during cytokinesis. The y-axis indicates percentage of cells, dark gray represents RhoA^+^ cells, light gray represents RhoA^−^ cells, the corresponding genotypes are indicated below and the error bars represent the standard deviation; * indicates *p*-values of 0.00383 for RhoA^+^ cells in wt/PKCε^-/-^ and 0.00471 in wt/PKD3^-/-^ by *t*-test. (**B**) Wild-type and PKD3-deficient MEFs were assessed for RhoA activity using an activated RhoA pull down following nocodazole treatment (0, 1 and 3 h).

RhoA has been identified as a key player during cytokinesis [14] and has recently been placed downstream of a PKCε-mediated signalling cascade [11,15]. Based on our data suggesting that PKD3 acts downstream of PKCε during cytokinesis, we investigated whether PKD3 deficiency could alter RhoA localisation and/or activity. First, we used an antibody-based immunofluorescence approach to determine whether PKD3/GFP and RhoA localisation correlate during cytokinesis in wild-type MEFs. RhoA was shown to localise to the equatorial cortex at an early stage of cytokinesis, with no overlap with PKD3/GFP staining (Figure 4B, first row). Once a cleavage furrow was established, RhoA co-localised with PKD3/GFP at the furrow (Figure 4B, second row). During cytokinesis progression, RhoA was shown to co-localise with the broader phospho-PKD signal at the midbody (Figure 4C, first row). Finally, the activated PKD3 signal appeared to flank but not overlap with RhoA on either side of the future daughter cells (Figure 4C, second row). A similar midbody staining pattern for RhoA was also observed in PKCε- and PKD3-deficient MEFs (Supplementary Figure S2), except that phosphorylation of PKD3 was not detectable. During this analysis, we found that MEFs deficient for PKCε or PKD3 had a higher percentage of cells with a RhoA signal at the midbody than wild-type MEFs under normal growth conditions. While 54% of wild-type MEFs showed a RhoA^−^-positive midbody (RhoA^+^) during cytokinesis (estimated by an established cleavage furrow), 46% of cells lacked RhoA staining (RhoA^−^). In contrast, PKCε- and PKD3-deficient MEFs showed a shift in this distribution to 80% RhoA^+^ and 20% RhoA^−^ cells (see calculation in Figure 5A), suggesting that the RhoA protein can be maintained in this position. We hypothesised that persistent midbody localisation may be associated with a prolonged RhoA activation status. To test this possibility, we performed a Rhotekin-RBD pull-down assay and showed that in wild-type MEFs treated with nocodazole (NC) for 12–14 h, RhoA activity peaked at 45–60 min after NC release and returned to basal levels by 3 h (Figure 5B, left panel). In contrast, RhoA activity was prolonged for up to 3 h after NC release in PKD3-deficient MEFs (Figure 5B, right panel). In conclusion, we show that PKD3 localises in close proximity to RhoA during cytokinesis and is required to regulate RhoA activity but not its localisation.

## 4. Discussion

In this report, we describe an isoform-specific, non-redundant function of PKD3 in the regulation of RhoA activity during cytokinesis. Using genetically depleted MEFs, we show that the absence of PKD3 results in an abscission defect, leading to an increase in double/multi-nucleated cells. A very similar phenotype has previously been described for PKCε [11], so one of our aims was to determine whether the predicted nPKC/PKD signalling axis was also relevant in this context. Indeed, we showed that PKCε is required to activate PKD3 during cytokinesis, based on the following data: (i) a dominant active PKD3 version mimicking activation loop phosphorylation was able to rescue the PKCε phenotype; (ii) a dominant negative PKD3 version mimics the double/multi-nucleus phenotype of PKCε and PKD3 deficiency in wild-type MEFs; and (iii) specific localisation and activation loop phosphorylation of PKD3 during cytokinesis is lost in a PKCε-deficient background. Prior to this study, very few reports have demonstrated distinct PKCε/PKD3 signalling events in different cell contexts and signalling pathways [16,17]. To our knowledge, this work suggests a novel function of the PKCε/PKD3 signalling axis in cytokinesis. Interestingly, deficiency of both PKCε and PKD3 in primary MEFs did not show any differences in cytokinesis compared to wild-type MEFs. However, once the MEFs had senesced and become immortalised, both mutant cell lines showed a significant increase in the number of double/multi-nucleated cells compared to corresponding wild-type MEFs of the same genetic background. The fact that this phenotype occurs after immortalisation may explain why PKD3^-/-^ mice do not show a severe cytokinesis-related phenotype, which is also observed in PKCε^-/-^ mice. Notwithstanding the fact that accumulating evidence has helped to explain in general terms how immortalisation works in murine fibroblast cells [12], a clear statement regarding the role of PKD3 (and PKCε) during cytokinesis after immortalisation is not possible. Also, in a recently published transcriptome study analysing primary versus immortalised RNA data from PKD3 wild-type and null MEFs, no pathways were identified that provide a direct mechanistic link to cytokinesis [18]. One possible explanation is that multiple regulatory pathways coexist in primary cells, which may be restricted after immortalisation, and that a PKD3-dependent pathway becomes dominant after immortalisation, leading to the observed phenotype in PKD3-deficient cells. Our results provide new insights into the spatial and temporal localisation of PKD3 during cytokinesis. While we were unable to localise PKD3/GFP to the equatorial cortex, once a cleavage furrow is established, PKD3/GFP co-localises to the actomyosin ring. As cytokinesis progresses, PKD3/GFP becomes concentrated in the midbody region. Interestingly, activated PKD3 (as monitored by phosphorylation of the activation loop) is only detectable when localised to the midbody, suggesting a functional role at this later stage. Given our results using the PKD3 activation loop mutants, we predict that PKCε is the activating kinase of PKD3 in the context of cytokinesis. PKCε has been previously localised to the cleavage furrow [11] and has been predicted to function upstream of PKDs in previous reports [8]. Our study supports these findings and fills a gap in the predicted signalling of PKCε to RhoA [11,15]. The small GTPase RhoA plays a central role in the establishment of the cleavage furrow [10], supported by its specific localisation during cytokinesis [19]. Building on the previous finding that RhoA localises to the equatorial cortex and cleavage furrow, we identified RhoA at a late stage of cytokinesis as a single spot located at the midbody (see Figure 4C). We hypothesised that this spot represents a pool of GTP-bound RhoA that is actively involved in actin polymerisation and myosin recruitment [20,21]. To facilitate depolymerisation of the actomyosin ring towards abscission, active GTP-bound RhoA must be converted to its inactive GDP-bound form, resulting in its release from the furrow. Our data provide evidence that PKD3 is involved in this process because (i) activated PKD3 co-localises or is in close proximity to RhoA at the midbody and (ii) PKD3-deficient MEFs show an increased number of cells with a RhoA midbody localisation. We interpret the latter finding as an indication that activated RhoA is maintained in this position. Two possible circumstances could be responsible for this. First, the GTP-bound (i.e., active) state of RhoA could be caused by an overactive guanine nucleotide exchange factor (GEF) or a non-active GTPase-activating protein (GAP). Both groups of proteins are potential PKD3 substrates, supported by in-house in silico analysis of the potential phosphorylation motifs and colocalisation within a cell, as studied by a number of research groups. although neither has been characterised as such so far. Indeed, when we analysed the amount of active GTP-bound RhoA during cytokinesis, it became clear that PKD3 deficiency mediated prolonged RhoA activity after NC release. Thus, we speculate that due to the increased residence time of RhoA at the cleavage furrow, the abscission process is more frequently not completed in mutant MEFs than in wild-type MEFs, resulting in bi- and multi-nucleated cells. The PKCε/PKD3/RhoA signalling axis we postulate cannot be easily integrated into the established signalling pathways that regulate cytogenesis (reviewed in [22,23]) and thus has a certain uniqueness. For PKCε, it has been shown that in addition to the activation of PKD3 that we have demonstrated, there are other functions in the context of cytogenesis, such as activating phosphorylation of Aurora B, which appears to be independent of PKD3 [24]. In principle, it cannot be excluded that PKD3 also has multiple functions in this context and is involved in several signalling pathways similar to PKCε. However, based on the current data, any statement in this regard would be purely speculative. Deregulated cytokinesis can have a major impact on carcinogenesis [25]. To date, PKD3 has been identified as a key player in prostate cancer cell invasion [26]. In addition, Olayioye and coworkers [27] showed that PKD3 promotes the proliferation of triple negative breast cancer cells through the mTORC1-S6K1 pathway. Only one report [16] has implicated the PKCε/PKD3 signalling axis downstream of the Akt and Erk1/2 oncogenic signalling pathways in the growth and survival of prostate cancer cells. None of these reports have suggested a potential function of PKD3 in cytokinesis. The in vivo relevance of our findings in the context of tumour initiation and progression will be tested in the future by crossing the conditional PKD3 allele with defined murine tumour models. This will allow us to assess whether the cytokinesis dysfunction mediated by PKD3 deficiency also plays a role in driving tumour development in vivo.

## 5. Conclusions

Taken together, our study provides the first evidence that PKD3 is functionally involved in cytokinesis regulation and defines PKD3 as a downstream kinase of PKCε-mediated signalling to RhoA in this context. Our initial observation is limited to immortalised MEFs, which raises the question of how relevant this phenotype is to a wild-type cell. However, we believe that it is relevant in the context of tumourigenesis, which ultimately results from immortalisation. The extent to which GFP and similar overexpressed constructs reflect the in vivo function of individual proteins can only be verified by alternative experiments using other tools, if available. Further future experiments aiming to identify the corresponding PKD3 substrates could consist of a combination of pull-down experiments followed by proteomic analysis (as performed, for example, by Capalbo et al. [28]). Certainly, future identification of interacting proteins and/or substrates will help to further elucidate the mechanistic role of PKD3 during cytokinesis that we have identified.

## Figures and Tables

**Table 1:** PCR primer sequences for genotyping.

Primer ID	Primer Sequence
a	GACTGTCATCACCAGCATCTTTCAGC
b	CCTGGAGAGAGACTGAAGCCTTGG
c	GCAGTGGCTGATCATGTATTGAGCAG
d	CTGACAGGACAACTTCTACCAGGTC
e	GCCACACTGTACCCCAGCTCATG
f	GGGTAGAGCGCTCTTCACAGAG

**Table 2:** PKD3 mutagenesis primer.

Primer ID	Primer Sequence
PKD3.DN, forward	CATCATTGGTGAGAAGGCATTCCGGAGGGCAGTGGTAGGAACTCC
PKD3.DN, reverse	GGAGTTCCTACCACTGCCCTCCGGAATGCCTTCTCACCAATGATG
PKD3.CA, forward	GCATCATTGGTGAGAAGGAGTTCCGGAGGGAAGTGGTAGAACTCCAG
PKD3.CA, reverse	CTGGAGTTCCTACCACTTCCCTCCGGAACTCCTTCTCACCAATGATGC

## Data Availability

Data are contained within the article and supplementary materials.

## Supplementary Materials

The following supporting information can be downloaded at https://www.mdpi.com/article/10.3390/biomedicines13020345/s1, Figure S1. Targeting the PRKD3 locus in mice; Figure S2. Localisation of active PKD/GFP and RhoA in MEFs of various genetic backgrounds.
