# Supplementary material for: A Protein Kinase Cε/Protein Kinase D3 Signalling Axis Modulates RhoA Activity During Cytokinesis
Source: Biomedicines. 2025 Feb 3;13(2):345. doi: 10.3390/biomedicines13020345 (PMC11853137; doi:10.3390/biomedicines13020345)
Supplement: Supplementary file 1 [file biomedicines-13-00345-s001.zip › biomedicines-3403530-supplementary.pdf]

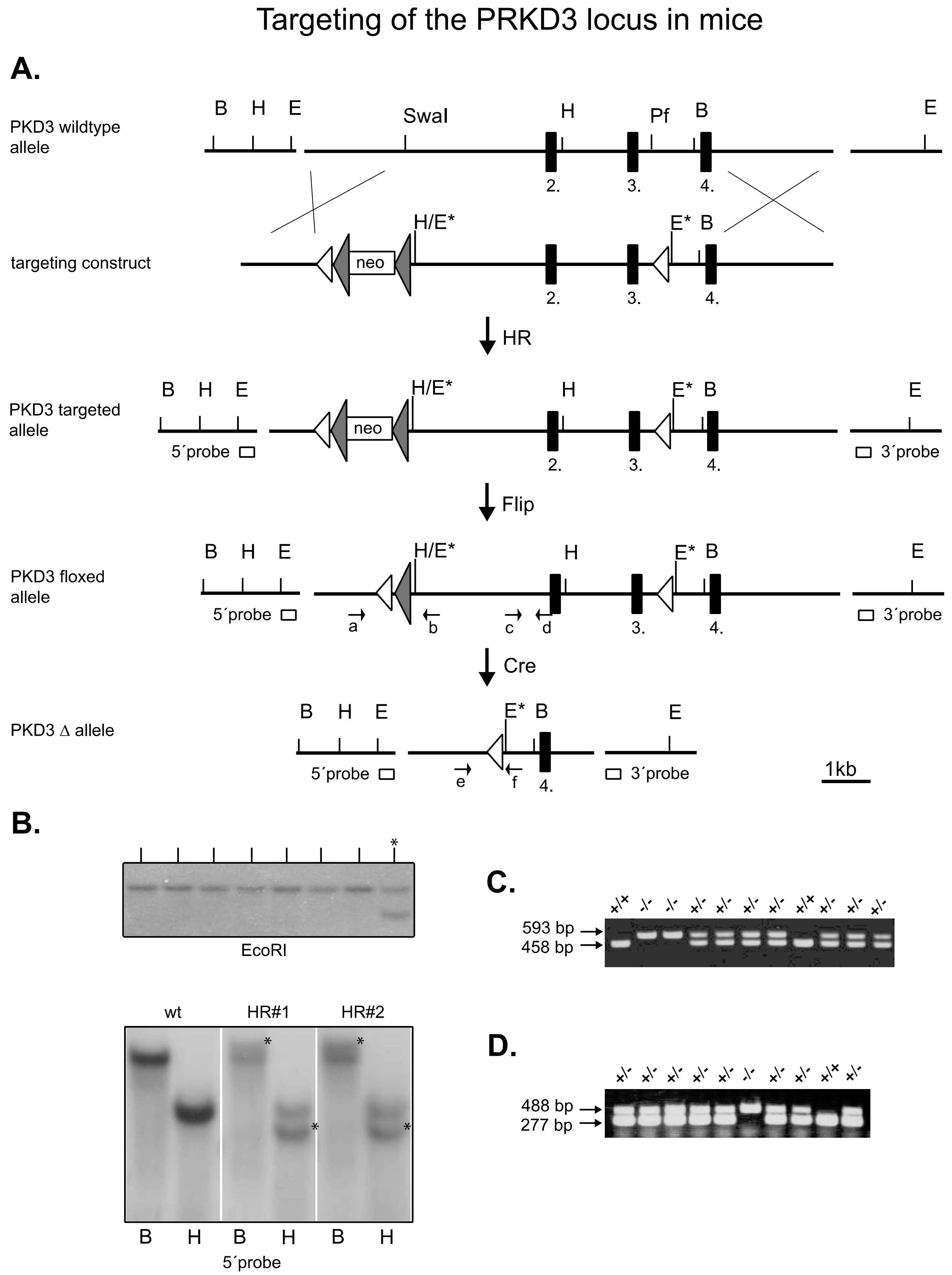

Figure S1. *Targeting the PRKD3 locus in mice.* A) A schematic representation of the chosen gene targeting strategy is shown. The relevant restriction sites are indicated (B, BamHI; E, EcoRI; H, HindIII; Pf, PflMI and SwaI). The black boxes represent the 2nd, 3rd and 4th exon of the PRKD3 gene. The white boxes represent the PCR-amplified DNA probes (5′- and 3′- probe) used for the Southern blot analysis. The open triangles are loxP sites, and the filled triangles represent FRT sites. The top shows the wild-type PRKD3 gene locus, part of which was chosen for targeting, followed below by a schematic view of the targeting vector containing a single loxP site 3′ of the 4th exon and an inserted neomycin cassette 5′ of the 2nd exon. The targeted PRKD3 allele represents the PRKD3 gene

locus after homologous recombination with the targeting vector, which gave rise to the PRKD3 floxed allele shown after crossing to a ubiquitously expressing Flp mouse line. Subsequently, the PRKD3 floxed allele was crossed to a mouse line with ubiquitous Cre expression, generating the PRKD3 Δ allele shown at the bottom. B) Representative Southern blot screen of G418-resistant ES cell clones after electroporation of the targeting vector. An EcoRI digest identifies a wild type 18.6-kb genomic DNA fragment (present in all lanes) and a recombinant 4.5-kb genomic DNA fragment (lane marked with asterisk) using the 5′ probe. Below a representative Southern blot characterization of recombinant ES cell clones (HR#1 and HR#2) compared to wild type (wt) is shown. Again the 5′ probe was used in combination with BamHI (B) and HindIII (H). Recombinant genomic DNA fragments are indicated with an asterisk: B; 11.3-kb - wild type and 13.4-kb - recombinant, H; 6.4-kb - wild type and 5.2-kb - recombinant. C) Representative PCR genotyping of one litter generated from crossing PRKD3$^{flox/+}$ mice. The primer pair a/b amplifies either a 458-bp wt DNA fragment or a LoxP-specific DNA fragment of 593 bp. The flox genotypes are indicated above (+/+; +/-: -/-). D) Representative PCR genotyping of one litter generated from crossing PRKD3$^{\Delta/+}$ mice. The primer pairs used amplify either a 277-bp wt fragment (c-d) or a deletion-specific fragment of 488-bp (e-f). The genotypes are indicated above (+/+; +/-: -/-).

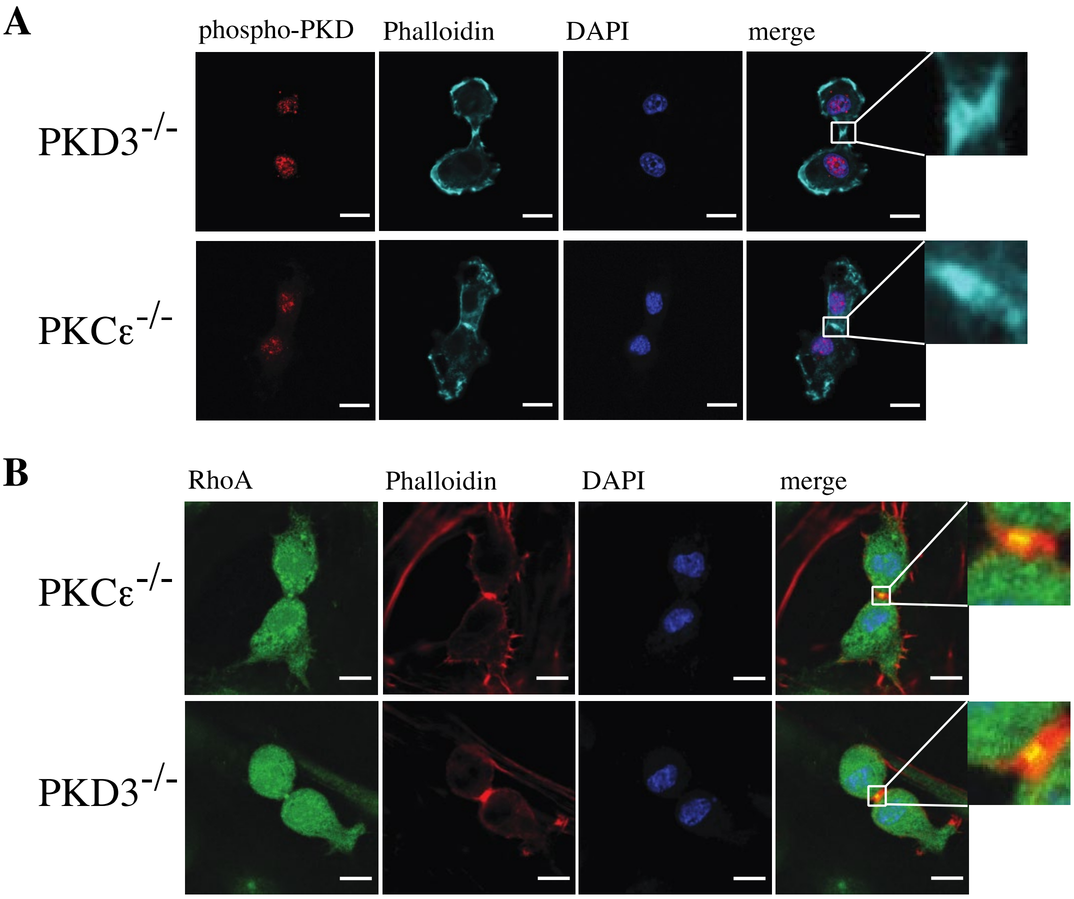

Figure S2. *Localization of active PKD/GFP and RhoA in MEFs of various genetic background:* A) MEFs of indicated genotype were fixed with 4% PFA and co- stained with an anti-phospho Ser730/734 PKD antibody and DAPI at later stages of cytokinesis. In each case, a phospho-PKD signal was visible at the newly formed nucleus, but no signal was detected at the cleavage furrow. Higher magnification inserts represent the furrow area of the corresponding merge as indicated. B) MEFs of indicated genotypes were co-stained with anti- RhoA and phalloidin. Both samples show that RhoA localization at the cleavage furrow does not changed irrespective of the mutant background. scale bar, 24 μm.
